# Ultrasensitive tapered optical fiber refractive index glucose sensor

**DOI:** 10.1038/s41598-023-31127-4

**Published:** 2023-03-18

**Authors:** Erem Ujah, Meimei Lai, Gymama Slaughter

**Affiliations:** 1 Center for Bioelectronics, Old Dominion University, Norfolk, VA 23508 USA; 2 Department of Electrical and Computer Engineering, Old Dominion University, Norfolk, VA 23508 USA

**Keywords:** Biochemistry, Optics and photonics

## Abstract

Refractive index (RI) sensors are of great interest for label-free optical biosensing. A tapered optical fiber (TOF) RI sensor with micron-sized waist diameters can dramatically enhance sensor sensitivity by reducing the mode volume over a long distance. Here, a simple and fast method is used to fabricate highly sensitive refractive index sensors based on localized surface plasmon resonance (LSPR). Two TOFs (*l* = 5 mm) with waist diameters of 5 µm and 12 µm demonstrated sensitivity enhancement at λ = 1559 nm for glucose sensing (5–45 wt%) at room temperature. The optical power transmission decreased with increasing glucose concentration due to the interaction of the propagating light in the evanescent field with glucose molecules. The coating of the TOF with gold nanoparticles (AuNPs) as an active layer for glucose sensing generated LSPR through the interaction of the evanescent wave with AuNPs deposited at the tapered waist. The results indicated that the TOF (Ø = 5 µm) exhibited improved sensing performance with a sensitivity of 1265%/RIU compared to the TOF (Ø = 12 µm) at 560%/RIU towards glucose. The AuNPs were characterized using scanning electron microscopy and ultraviolent-visible spectroscopy. The AuNPs-decorated TOF (Ø = 12 µm) demonstrated a high sensitivity of 2032%/RIU toward glucose. The AuNPs-decorated TOF sensor showed a sensitivity enhancement of nearly 4 times over TOF (Ø = 12 µm) with RI ranging from 1.328 to 1.393. The fabricated TOF enabled ultrasensitive glucose detection with good stability and fast response that may lead to next-generation ultrasensitive biosensors for real-world applications, such as disease diagnosis.

## Introduction

One of modern medicine's biggest challenges is developing cost-effective technologies that can diagnose a disease in a timely and accurate manner and unaffected by electromagnetic interference (EMI). The label-free optical sensor offers a promising approach to biochemical sensing in nearly any environment, including those with EMI^1–3^. Most binding events, such as DNA hybridization, antibody-antigen recognition, chemical reactions, and changes in concentration, usually lead to changes in the optical sensor's surrounding environment and are known to change the sensing environment refractive index (RI). Moreover, this change in RI can quantitatively reflect the biosensors' detection ability. Therefore, measuring the small changes in RI that may result from a biochemical process is critical for biomarker detection^4,5^. Fiber optic-based RI sensors are characterized as label-free, with a variety of configurations. The most common fiber-based RI sensor are the Bragg grating (FBG) structures^6^, long-period gratings (LPG) forming a Mach–Zehnder interferometer^7^, micro-interferometers based on chemical etching^8^, and microstructured fibers^9^, and taper optical fiber^10^.

The material of the optical fiber is usually silica, which is non-toxic, eco-friendly, and much more resistant to corrosion than most materials, thus being a good candidate for sensing in harsh environments. Optical fibers provide excellent sensing versatility where they can be decorated with various materials (polymers, nanomaterials, etc.) or simply by changing the fiber configuration (interferometer, optrode, whispering gallery mode, etc.). Recently, tapered optical fibers (TOF) have attracted significant attention due to their ease of fabrication and enhanced optical properties^11–13^. TOF produces remarkably high optical intensity over a long distance, from several millimeters to a few centimeters. The long interaction length and high intensity of the TOF can enhance the light-matter interaction, thereby increasing the sensor’s sensitivity. Although there are various approaches to fabricating TOFs, as illustrated in Table 1, the flame brushing method is the most used, wherein a single mode or multimode glass optical fiber with the cladding removed is heated at the center of the fiber while simultaneously stretching the fiber at both ends to produce a symmetrical “waist” in the fiber^10^. This is the most straightforward and low-cost approach to fabricating a TOF.

**Table 1:** Tapered optical fiber fabrication methods.

Fabrication method	Advantages	Disadvantages
Flame brushing^10^	Simple way and low-cost Varying lengths and diameters	Lack of uniformity of applied heat Rough surfaces/insertion loss
Laser processing^8^	Sub-micron dimensions Smooth surfaces	Requires specialized equipment Insertion loss
Electrical heating^9^	No turbulence in comparison to flame brushing	Non-even heating distribution
Chemical etching^10^	High-quality Highly reproducible	Precision in hydrofluoric acid: time ratio Limited control over the shape

Furthermore, TOFs have been demonstrated to contain less than 100 photons at a time in the interaction (tapered) region using ultralow power level nonlinear spectroscopy^14^. This low dose of radiation also can reduce the risk of sample damage in the sensing environment. Another key advantage to using TOF is that they are very steady over time and can self-clean via fluid motion^15^. TOF systems have been reported for biochemical sensing^16–23^. In addition, the functionalization of nanoparticles on TOF has been reported to enhance fiber optic-based RI sensor’s property by creating localized surface plasmonic resonance (LSPR) that engages the electrons in the nanoparticles to oscillate coupling to the evanescent wave resulting in a significant change in refraction index^24^. Different types of nanoparticles, such as gold and silver^25,26^, magnetic nanoparticles^27,28^, carbon-based nanoparticles^29,30^, latex nanoparticles^31^, and liposome-based nanoparticles^32^ have been reported to enhance the surface plasmonic resonance signal for target analyte detection. AuNPs are widely used for functionalizing  TOF because of their strong induction of surface plasmonic resonance and their biocompatibility. Additionally, AuNPs are stable and resistant to oxidation, making them more durable for long-term applications. Lin et al.^17^ demonstrated a localized surface resonance TOF sensor using AuNPs. A waist diameter of 48 μm and length of 1.25 mm was achieved and then decorated with AuNPs. A sensitivity of 380%/RIU with RI ranging from 1.333 to 1.403 was reported. Tai and Wei^33^ demonstrated an intensity sensitivity of up to 8000%/RIU using a tapered fiber tip to enhance the sensitivity of optical fiber sensors further due to the nanometer tip. The tapered fiber refractive index sensors show excellent sensitivity with a fast response time in the local change of index of refraction in a real-time manner, which has enormous potential for label-free biosensing.

Research efforts with TOF-based RI sensors have resulted in devices with varied sensitivity and reproducibility. To overcome this limitation, this paper examines varying taper waist diameters with a long waist length and the coupling of AuNPs at the tapered waist region to harness the evanescent field for enhancing the efficacy of the light coupling and, thus, the sensitivity. Two TOF RI sensors with a more extended waist length (*l* = 5 mm) and smaller waist diameters of 5 µm and 12 µm were fabricated for glucose sensing. The waist diameter is critical in enhancing the sensitivity of TOF. The TOF (Ø = 5 µm) exhibits significantly improved glucose sensing capability in comparison to the TOF (Ø = 12 µm). However, the TOF (Ø = 12 µm) can be easily fabricated and is highly durable. AuNPs were prepared and used to decorate the surface of the TOF (Ø = 12 µm) for sensitivity enhancement. A power of less than 100 nW was used for the RI detection of glucose. This ultra-low power sensing provided experimental evidence for sensitive TOF RI detection at longer wavelengths (1559 nm) with a sensitivity of 2032%/RIU toward glucose with a linear RI range of 1.328–1.393. This results from the unique characteristics of AuNPs, such as their optical absorption and specific surface area, to enhance the light-matter interaction. The detection at the longer wavelength showed excellent sensitivity in the as-fabricated LSPR and other reported SPR systems^34,35^. TOF sensors can serve as promising candidates for non-invasive glucose monitoring in healthcare and the bioprocessing industries^36–39^.

## Results and discussion

**Figure 1:**
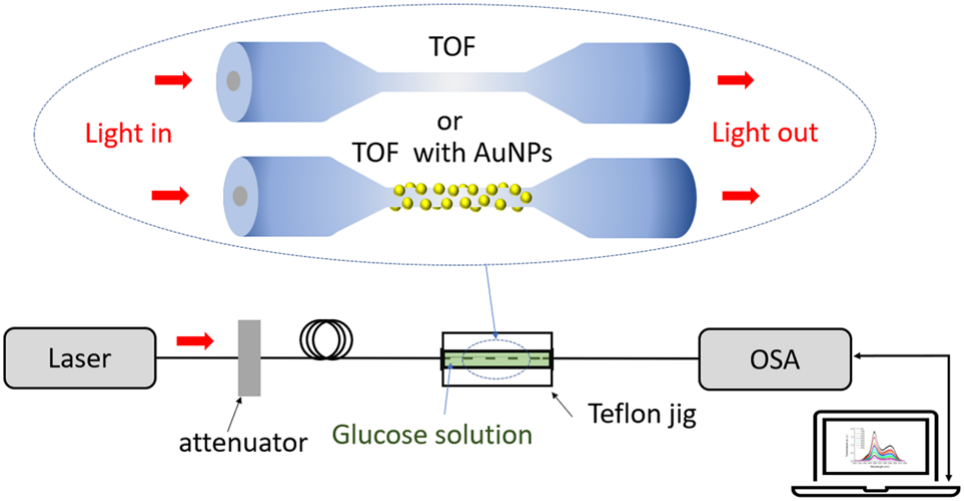
Experimental setup for glucose sensing.

The experimental setup is shown in Fig. 1. The TOF sensing region was suspended above the canyon floor and submerged entirely in the target analyte. The input light (Thorlabs ASE-FL7002 White Light Test Source, 1530–1610 nm) propagated through the TOF through an optical attenuator and polarization controllers. The light output was coupled to an Optical Spectrum Analyzer (Thorlabs OSA203C Fourier Transform Optical Spectrum Analyzer, 1.0–2.6 µm). The TOF comprised a transition region with a smooth linear taper profile and a small uniform waist diameter (Ø = 5 µm or Ø = 12 µm). A series of aqueous glucose solutions were prepared with mass ratios ranging from 0 to 45 wt%. The corresponding RI at 1559 nm was obtained.

### UV–Vis spectroscopy and structure of AuNPs

**Figure 2:**
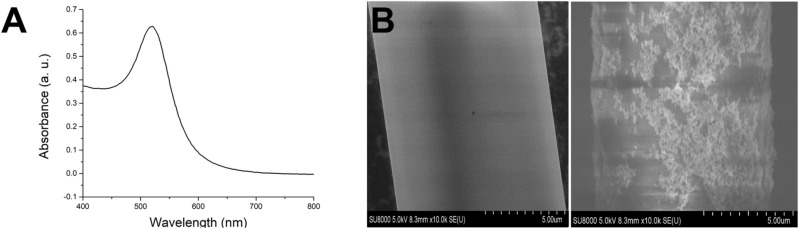
(**A**) UV–Vis spectra of the synthesized gold nanoparticles (AuNPs). (**B**) Scanning electron microscopy (SEM) of bare and AuNPs-decorated TOF.

Figure 2A shows the UV–Vis absorption spectrum of the citrate-capped AuNPs in water. During the AuNPs synthesis, citrate reduces Au(III) to Au. The AuNPs exhibit a strong absorption peak at 524 nm. The absorbance peak suggests that AuNPs are approximately 24–33 nm in size and agrees with earlier reports^40–42^. Figure 2B show the scanning electron microscopy (SEM) image of the bare TOF and AuNPs-decorated TOF RI sensor to confirm the chemisorption of AuNPs.

### Glucose sensing

**Figure 3:**
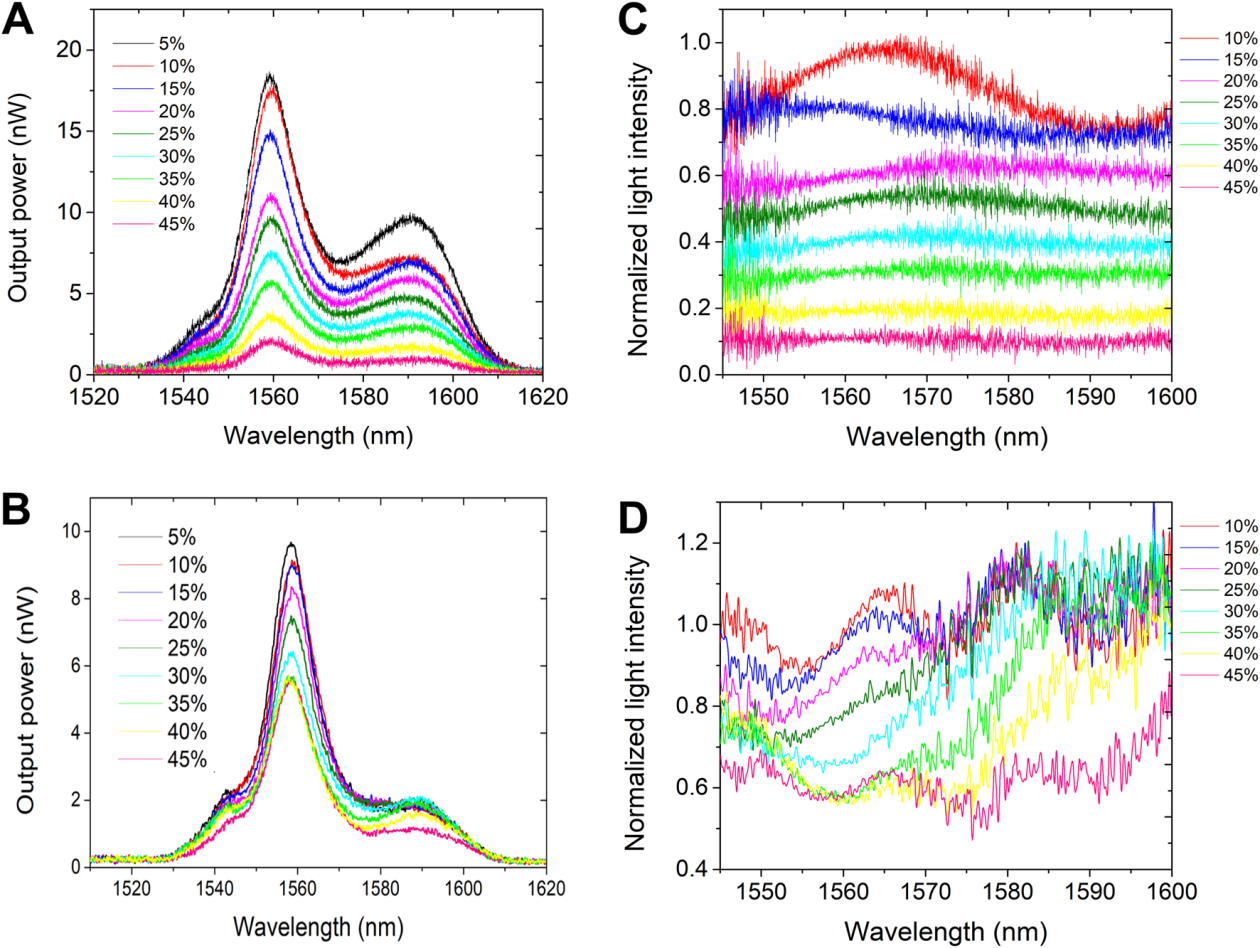
Optical spectra of bare TOF sensors in different mass fractions of glucose solutions. (**A**) TOF (Ø = 5 μm diameter) and (**B**) TOF (Ø = 12 μm diameter). (**C**,**D**) Corresponding normalized intensity spectra.

The comparative analysis of the optical spectra data indicates that the two TOFs with waist diameters of 5 µm (Fig. 3A) and 12 µm (Fig. 3B) impart the power output of the RI sensor. The power output response for the bare TOFs is observed across the near-infrared (NIR) wavelength range of 1520–1630 nm. Upon exposure to various glucose concentrations (5–45 wt%), the power output response decreases with the increase in glucose concentrations. The observed trend agrees with previous reports for other biomolecules^40,41^. The optical spectra were processed by integrating the area under the power output curve to generate the corresponding normalized power intensity curves depicted in Fig. 3C,D. At the peak wavelength of 1559 nm, the decrease in intensity can be clearly observed for each glucose concentration. No insignificant change was observed in the spectra after changing the light's polarization. This indicates that the TOF RI sensing is polarization insensitive, making it more practical^43^.

**Figure 4:**
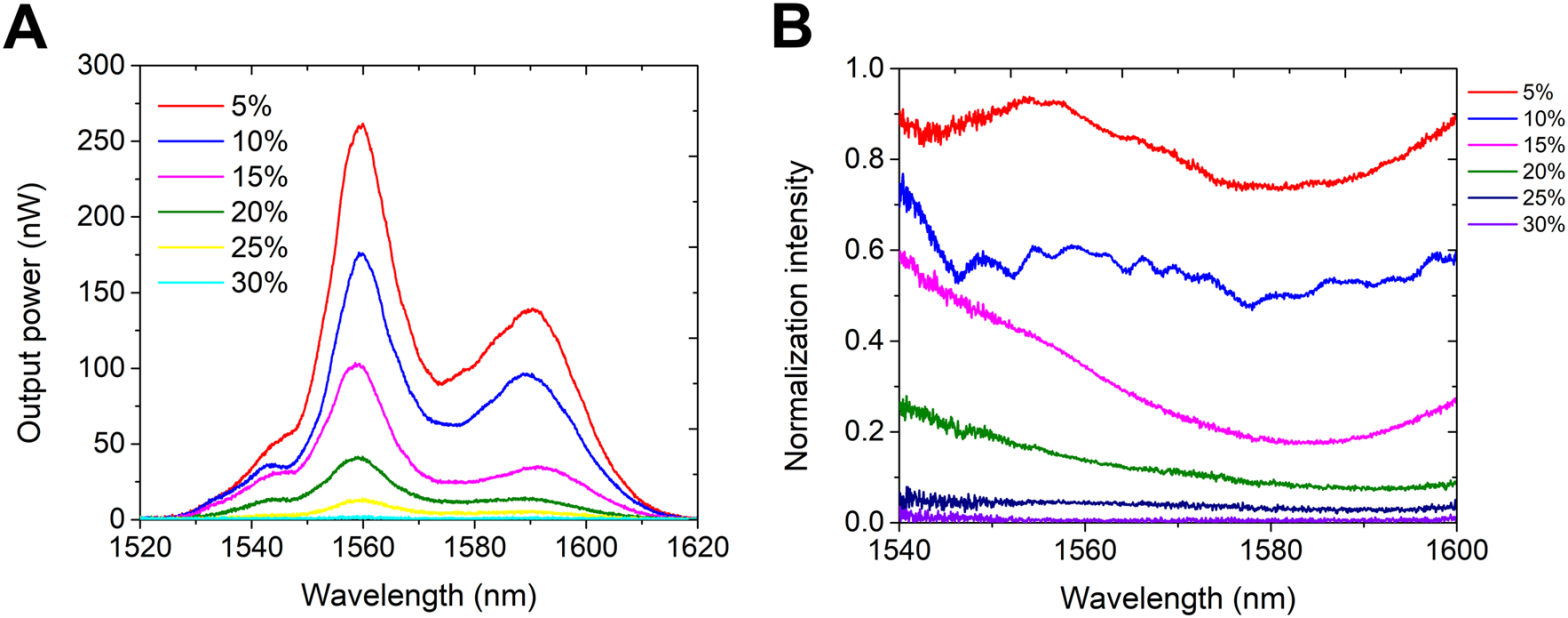
(**A**) Optical spectra of AuNPs decorated TOF ( Ø = 12 μm diameter) sensor in different mass fractions of glucose solutions and (**B**) Corresponding normalized intensity spectra.

**Figure 5:**
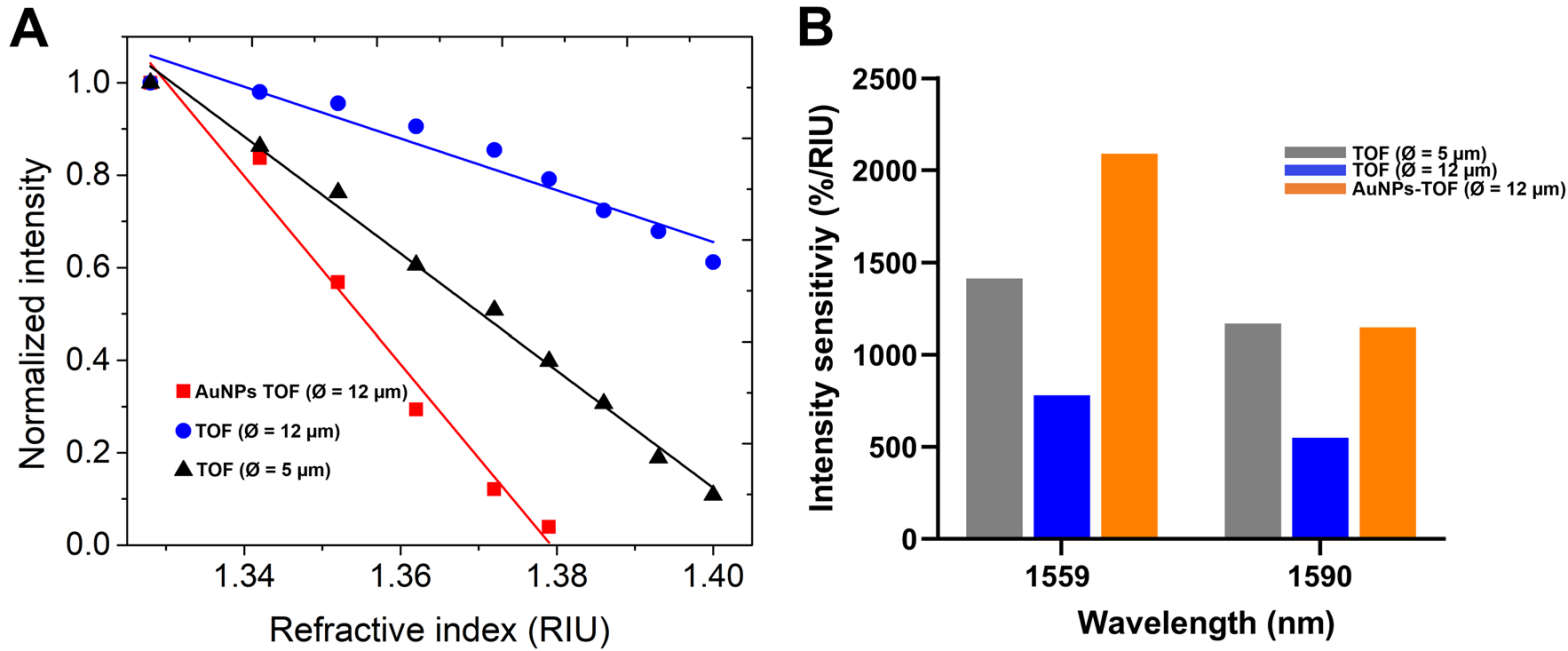
(**A**) Power intensity changes against refractive index of bare TOFs (red and blue) and AuNPs decorated TOF at *λ* = 1559 nm. (**B**) Comparison of sensitivity of TOF RI sensors.

The TOF with a larger waist diameter exhibited better reliability with reduced sensitivity. Therefore, to improve the sensitivity, AuNPs were coated on the surface of the fiber waist. From the AuNPs-decorated TOF (Ø = 12 µm) spectra (Fig. 4A), it is observed that the sensor's initial output in glucose is high due to the AuNPs coating. This is attributed to the inherently high surface area of the AuNPs and the LSPR, which enhance the interaction of glucose molecules with the sensing region, thereby dramatically altering the evanescent field. The enhanced evanescent field penetration depth is modulated by the RI and enables a more intense power output than the bare TOFs. As the RI decreases, the surface evanescent wave penetration depth decreases, resulting in little interaction between the evanescent field and the glucose molecules and little power output in the detection spectrum. The corresponding normalized power intensity curves in Fig. 4B show that the AuNPs coating significantly enhances the sensitivity of the TOF RI sensor and further depicts the occurrence of glucose molecule interaction with the sensing region. Figure 5 shows the calibration curve for the bare TOF and the AuNPs-decorated TOF sensors. The smaller waist diameter TOF (Ø = 5 µm) exhibited a sensitivity of 1265%/RIU compared to that of 560%/RIU for TOF (waist Ø = 12 µm) at λ = 1559 nm, as shown in Fig. 5A. The higher sensitivity towards glucose was observed because of the enhanced evanescent field interactions with glucose achieved with the smaller waist diameter TOF. The intensity sensitivity of the TOF sensor is given by^33^:

$$\mathrm{S}=\frac{\Delta I/{I}_{0}}{\Delta n}\times 100(\%/RIU).$$

A sensitivity of 2032% RIU at λ = 1559 nm was observed for the AuNPs-decorated TOF (Fig. 5A), representing an increase by a factor of ~ 4 compared to the bare TOF. The AuNPs generate an LSPR signal with high sensitivity to detect slight changes in the reaction. However, the AuNPs-decorated TOF sensor saturated at 30 wt% glucose, wherein the peak at 1559 nm disappears. This may result from glucose molecules adsorbing onto the sensing region. Overall, the AuNPs-decorated TOF generated more considerable power output changes than bare TOF. The sensitivity enhancement spans a broad spectrum range (1540–1610 nm) and is uniform. Additionally, the optical performance of the as-fabricated TOF sensor was compared with other optical fiber-based RI sensors tested in various analytes, as summarized in Table 2.

**Table 2:** Comparison of the analyte characteristic of optical fiber RI sensors.

Fiber type	Analyte	Material	Sensitivity	Linear range	Wavelength	References
TOF tip	Glycerin	bare	8000%/RIU	1.3–1.4	630 nm	^33^
Coreless silica fiber	Glucose	bare	1467.59 nm/RIU	1.364–1.397	1550 nm	^37^
Heterocore fiber	NaCl	Mxene	510.1%/RIU	1.3343–1.3765	500–1000 nm	^44^
Heterocore fiber	NaCl	bare	57.2%/RIU	1.3343–1.3765	500–1000 nm	^44^
U shape	Glucose	AuNPs, Glucose oxidase	2.899 nm/%; 5.101 dB/%	0.1–0.5%	1400–1600 nm	^45^
Unclad fiber	Sucrose	AuNPs, goat anti-rabbit IgG	13.09 AU/RIU	1.34–1.41	300–1000 nm	^46^
TOF	Glucose	Bare	1265%/RIU	1.328–1.393	1530–1610 nm	This work
TOF	Glucose	AuNPs	2032%/RIU	1.328–1.379	1530–1610 nm	This work

As the measured RI changed from 1.32 to 1.40, the change in light intensity was more significant, indicating that the AuNPs can produce sensitivity enhancement for TOF RI sensors. AuNPs also enable more contact areas for glucose molecules to interact at the sensor surface. A linear RI range of 1.34–1.40 (r^2^ = 0.9254) was observed for the TOF (Ø = 12 µm), and a wider linear range of 1.32–1.40 (r^2^ = 0.9940) was observed for the TOF (Ø = 5 µm). A similar linear range of 1.328–1.393 (r^2^ = 0.9781) was observed for the AuNPs decorated TOF and agrees with or exceeds recently reported fiber optic RI sensors^17,44^. A heterocore fiber exhibited an intensity sensitivity of 57.2%/RIU within a range of 1.334–1.377, and a MXene nanosheet-based heterocore fiber demonstrated a sensitivity of 510.1%/RIU with a range of 1.334–1.377^44^. Therefore, the AuNPs-decorated TOF RI sensor enhances the evanescent field to detect the RI change in the sensing environment, making the sensor ideal for glucose detection. Figure 5B compares the TOF RI sensors' sensitivity at 1559 nm and 1590 nm. These uniform sensitivities indicate that the AuNPs-decorated TOF RI sensor is a good option for biochemical detection at 1559 nm and 1590 nm.

In summary, we described and demonstrated the use of TOF as RI sensors for detecting various glucose concentrations. As the glucose concentration increased from 5 to 45 wt%, the sensor's power output intensity decreased consequently. Additionally, the bare TOF's sensitivity is highly related to its diameter and insensitive to light polarization, which is ideal for real-world applications. The bare TOF (Ø = 5 µm) sensor demonstrated better sensing capabilities than the bare TOF (Ø = 12 µm). We achieved an intensity sensitivity of 1265%/RIU over the RI range of 1.328–1.393. Upon decorating the TOF (Ø = 12 µm) with AuNPs, the sensor's sensitivity increased by ~ 4 times, although the linear RI range decreased slightly. The sensitivity was 2032%/RIU over the RI range of 1.328–1.379. The smaller the waist diameter TOF, the more sensitive the sensor was to changes in the biochemical environment. And the AuNPs decoration on the TOF surface resulted in sensitivity enhancement due to the high surface-to-volume ratio enabled by the AuNPs for biomolecule adsorption and the generation of localized surface plasmon resonance. The TOF (Ø = 12 µm) preparation method is simple, robust, reproducible, and can easily be decorated with nanomaterials to enhance sensing capability. However, the micron-sized tapered fiber sensor is exceptionally fragile, and its mechanical strength will need to be improved for real-world applications. Future work will explore ways to package the TOF RI sensor for detecting biorecognition events in biochemical processes.

## Methods

### Fabrication of tapered optical fiber

**Figure 6:**
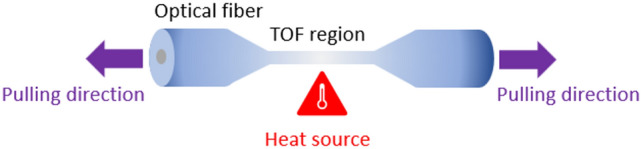
Schematic illustration of the heat and pull method for fabricating the tapered optical fiber.

The TOF was fabricated by the heat and pull method^45^. Briefly, the fiber coating on the two ends and the middle part of a standard commercially available optical fiber (Thorlabs1060XP, Single Mode Optical Fiber, 980–1600 nm, Extra-High Performance, Ø125 µm Cladding, Ø5.8 µm core) was carefully removed and cleaned with acetone. A propane air mixing flame was placed under bare fiber, as depicted in Fig. 6. The bare fiber ends were placed on fiber holders (Newport 125 µm Fibers, 561 Series) and pulled by two Aerotech Pro115SL linear stages controlled by A3200 Controllers to attain a sensing length of 5 mm. The transmission of the fiber was simultaneously monitored while pulling. The fabricated TOF was mounted in a custom-made canyon-shaped Teflon jig, and the sensing region was cleaned with acetone, followed by ultrapure deionized (DI) water rinse^13^. The canyon holds the sensing liquid (total volume = 2 ml).

### Gold nanoparticles synthesis and functionalization of TOF

**Figure 7:**
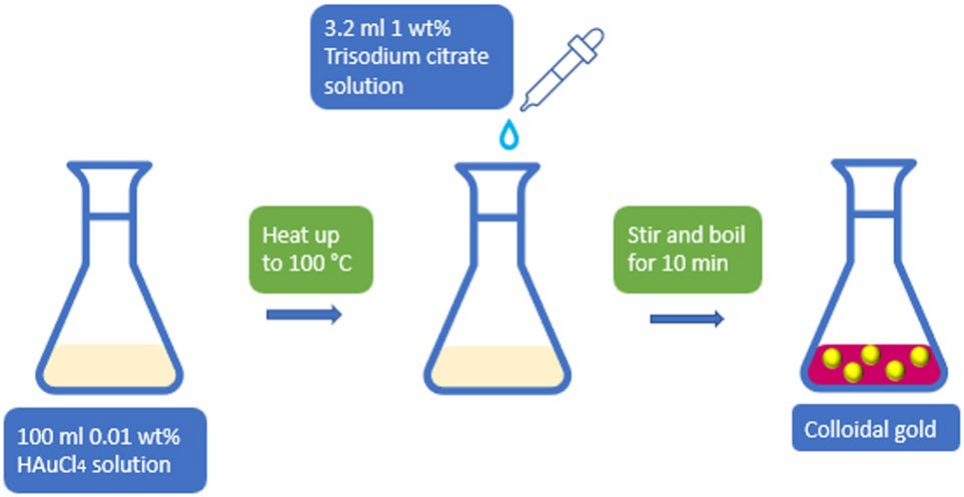
Gold nanoparticle (AuNPs) synthesis by sodium citrate reduction method.

As previously reported by Ma et al.^40^, the gold nanoparticles were synthesized using a chemical method, as illustrated in Fig. 7. All glassware was cleaned with freshly prepared aqua regia (HCl:HNO_3_, 3:1). The precursor solution was prepared using 0.01 g of gold (III) chloride trihydrate (HAuCl_4_·3H_2_O) dissolved in 100 mL DI water (18.2 MΩ cm) and brought to a boil under constant stirring with a magnetic stirrer on a hot plate. Immediately following boiling, 3.2 mL 1 wt% of trisodium citrate (Na_3_C_6_H_5_O_7_) solution was quickly added and stirred for 10 min. The Na_3_C_6_H_5_O_7_ serves as the reducing and capping agent in nanoparticle synthesis. The color of the solution changed from yellow to red wine during this period signifying the fast reduction of gold. The reaction solution was allowed to cool to room temperature. The obtained AuNPs were centrifuged and resuspended in DI water and stored at 4 °C when not in use. The UV–Vis spectra of AuNPs samples were recorded using UV–Vis absorption spectroscopy (Spectra Max M5 microplate reader, Molecular Devices, LLC.).

Afterward, the TOF fiber was functionalized by the salinization process method^47^. The sensing region of the TOF was cleaned with acetone, followed by a DI water rinse. After drying, the sensing region was immersed in freshly prepared piranha solution (H_2_SO_4_:H_2_O_2_, 3:1) for 30 min and thoroughly rinsed with DI water before drying at 90 °C for 30 min. Upon drying, the TOF was treated with 1% (v/v) solution of 3-aminopropyltriethoxysilane (APTES) in ethanol for 24 h at room temperature. The APTES functionalized TOF was rinsed with ethanol, followed by DI water rinse to remove any unbound APTES, and air dried. The functionalized TOF was incubated in 2 mL of the synthesized AuNPs solution for 8 h at room temperature to form a coating of AuNPs on the TOF sensing region. The AuNPs-decorated TOF was rinsed with DI water and used for RI sensing. The use of an organosilanes results in a very robust monolayer of metallic nanoparticles integration over the tapered optical fiber based on the chemisorption of nanoparticles^48^.

## Data Availability

The datasets used and/or analyzed during the current study available from the corresponding author on reasonable request.
